# Analysis of the Distribution of Magnetic Fluid inside Tumors by a Giant Magnetoresistance Probe

**DOI:** 10.1371/journal.pone.0081227

**Published:** 2013-11-29

**Authors:** Chinthaka P. Gooneratne, Adam Kurnicki, Sotoshi Yamada, Subhas C. Mukhopadhyay, Jürgen Kosel

**Affiliations:** 1 Computer, Electrical and Mathematical Sciences and Engineering, King Abdullah University of Science and Technology, Thuwal, Makkah, Kingdom of Saudi Arabia; 2 Automation and Metrology Department, Lublin University of Technology, Lublin, Poland; 3 Institute of Nature and Environmental Technology, Kanazawa University, Kanazawa, Ishikawa, Japan; 4 School of Engineering and Advanced Technology, Massey University, Palmerston North, Manawatu, New Zealand; University of California at Berkeley, United States of America

## Abstract

Magnetic fluid hyperthermia (MFH) therapy uses the magnetic component of electromagnetic fields in the radiofrequency spectrum to couple energy to magnetic nanoparticles inside tumors. In MFH therapy, magnetic fluid is injected into tumors and an alternating current (AC) magnetic flux is applied to heat the magnetic fluid- filled tumor. If the temperature can be maintained at the therapeutic threshold of 42°C for 30 minutes or more, the tumor cells can be destroyed. Analyzing the distribution of the magnetic fluid injected into tumors prior to the heating step in MFH therapy is an essential criterion for homogenous heating of tumors, since a decision can then be taken on the strength and localization of the applied external AC magnetic flux density needed to destroy the tumor without affecting healthy cells. This paper proposes a methodology for analyzing the distribution of magnetic fluid in a tumor by a specifically designed giant magnetoresistance (GMR) probe prior to MFH heat treatment. Experimental results analyzing the distribution of magnetic fluid suggest that different magnetic fluid weight densities could be estimated inside a single tumor by the GMR probe.

## Introduction

Hyperthermia therapy is a cancer treatment technique that uses heat to destroy tumors. Temperatures in the range of 42–45°C are known to kill cancer cells while having no, or minimal, effect on healthy cells [1]–[5]. The most common method of heating tumors is by electromagnetic radiation [6]. Two disadvantages of electromagnetic radiation are the inhomogeneous heating of tumor tissue and the heating of healthy tissues, due to the variation in the electrical properties of tissues. Inhomogeneous heating can result in under-treatment of a tumor; while heating of healthy tissues can cause burns, blisters and discomfort. Magnetic fluid hyperthermia (MFH) seeks to address these two issues by injecting magnetic nanoparticles into the tumor region, thereby selectively targeting tumor tissue and depositing heat in a localized manner [7]–[10]. The injected region is heated by the application of an alternating (AC) magnetic flux density. The energy absorbed from the AC magnetic flux is transformed to heat due to Neel relaxation and Brownian motion of the magnetic nanoparticles [7]. Such localized treatment, which results in very high spatial selectivity in the target region, cannot be achieved with radiation-based therapies because unwanted heating due to the electrical conductivity of healthy tissues cannot be avoided during radiation. Moreover, unlike radiation-based therapies, MFH can target deep-seated tumors since the penetration depth does not depend on the frequency.

The distribution of the magnetic fluid, once injected into a tumor site, depends on many factors, such as particle size, surface characteristics and the dosage of the injected magnetic fluid, heterogeneity of the tumor and surrounding tissue, size and pH of the tumor, blood flow in the tumor and surrounding areas, and the applied magnetic flux strength [2], [8], [11]–[15]. For effective MFH treatment, tumors must be heated uniformly [9], [10], [15]–[19]. Given that the applied magnetic flux density is uniform, the magnetic fluid injected into the affected area must also be uniform for homogenous heating of the tumor [20]–[24]. However, magnetic fluid injected into tumor sites can spread into neighboring tissue [25]–[27], which can lead to an inhomogeneous distribution of the fluid, and a decrease in the density of the magnetic fluid inside the tumor; hence, the relative permeability of surrounding, healthy tissue cannot be assumed to be 1. The application of an external AC magnetic flux density could then cause inhomogeneous heating of the tumor and possibly heat surrounding healthy cells, leading to possible necrosis of healthy tissue [28], [29]. However, the goal of MFH therapy is to protect healthy tissue from damage while destroying tumor cells [30]. Since the specific heat capacity generated is directly proportional to the density of the magnetic fluid, it is critical to check and confirm the distribution of the injected magnetic fluid [31]–[34].

The most common method of assessing and controlling temperature in MFH therapy is by the use of thermocouples or fiber-optical thermometers that are inserted by the surgeon into the tumor to measure the temperature [35], [36]. This method, while inexpensive, is not very accurate and requires magnetic resonance imaging (MRI) or computer tomography (CT) scans to locate the presence of magnetic fluid. MRI and CT scans are also directly used to estimate temperature, in a non-invasive manner, but these instruments are both bulky and expensive to use. Besides, large errors may be caused in the MRI due to uncertainty in the reference position which is caused by movement of the patient; from breathing/heartbeat to sudden involuntary movements. Several other methods that could be used to monitor temperature also have limitations. For instance, the density difference between bones and organs make it difficult for ultrasound to measure temperature. It is also difficult to integrate fluorescent and optical films into a surgical setup, and superconducting quantum interference devices, while being sensitive to minute magnetic fields, require large liquid helium cryostats for operation in addition to being expensive [37]. Moreover, using these methods during hyperthermia therapy might influence the temperature readout.

In this paper, we describe a giant magnetoresistance (GMR) probe designed to be inserted into the vicinity of the tumor tissue, in a minimally-invasive way, to analyze the distribution of the magnetic fluid inside the tumor. The distribution analysis is performed prior to the heating step in MFH therapy, at much lower magnitudes (<0.5 mT) and frequencies (<1000 Hz) than those typically used in MFH therapy (2–30 mT and 0.1–10 MHz). The analysis of the magnetic fluid distribution in the injected area allows the strength, frequency and localization of the applied external AC magnetic flux density needed to destroy the tumor to be determined without affecting healthy cells. Furthermore, the distribution analysis identifies if and where inside the tumor the magnetic fluid is inhomogeneous. In such a case, procedures such as multi-site injections can be used to increase the homogeneity of the magnetic fluid distribution inside the tumor [38]–[40]. Compared with the temperature measurement methods mentioned above, the method explored in this paper indirectly gives information about the temperature; the probe described here can be used to map the distribution of specific heat capacity, thus providing information about the temperature to assess the risks associated with the length of therapy and temperature elevation. It is also possible to use the probe to perform post-therapy analysis of the distribution of any remaining magnetic fluid at the site. Such information would allow the surgeon to determine how much more magnetic fluid would be required for ensuing therapy and also to monitor the fate of the magnetic fluid after treatment.

The probe proposed in this paper is designed to be small and lightweight. It must also be highly sensitive with excellent spatial resolution; critically important features when measuring very small changes in magnetic flux densities inside tumors. Moreover, the equipment necessary to process, read out and interpret the signals from the probe is simple and inexpensive. The probe has a fast response and can be operated continuously. Its maintenance cost is low; it is durable, stable and minimally invasive. The USB interface of the probe provides compatibility with standard interfaces; it can therefore pass seamlessly from engineering production to the operating theatre with minimal fuss.

## Materials and Methods

### 1. Analytical Basis for Estimating the Weight Density of Magnetic Fluid inside Tumors

One of the most important considerations in MFH therapy is the heat capacity required to damage or destroy cancer cells [41], [42]. Heat capacity, *Q* (W/ml), can be calculated as 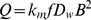
(1)where *k_m_* = 3.14×10^−3^ (W/Hz/(mgFe/ml)/T^2^/ml), *f* is the exciting frequency (Hz), *D_w_* is the weight density of the magnetic fluid (mgFe/ml) and *B* is the amplitude of the applied magnetic flux density (T). *k_m_* is a coefficient that depends on the properties of the magnetic fluid. The value for *k_m_* was obtained by experimentation with Resovist®, a clinically approved magnetic fluid that includes superfine iron oxide nanoparticles coated with carboxydextran.

In general, equation (1) can be used to determine the heat capacity for effective treatment unless the tumor is close to large vessels, in which case the “bio-heat” equation that takes into account heat depletion due to blood perfusion should be used [43]. Once injected into tumors, *D_w_* will depend on the retention of magnetic fluid by the affected cells. Magnetic fluid injected into a target site spreads to surrounding tissue, and also drains through blood vessels and lymph nodes. However, it must be noted that tumor cells generally absorb nine times more magnetic particles than normal cells, though this uptake may depend on several factors such as cell type and nanoparticle coating [44]. Several methods have been used to increase the retention of magnetic nanoparticles in affected cells, for example coupling magnetic nanoparticles to tumor-specific ligands such as antibodies, slow infiltration, and repeated multi-site injections [7].

Atkinson and Brezovich proposed a maximum limit on the product *H*×*f* = 4.85×10^8^ Am^−1^Hz based on patient discomfort nearly 20 years ago [45], [46]. In this product, *H* is the magnetic field in A/m and *f* is the frequency in Hz. Their test was based on the patient withstanding the treatment for more than one hour without any major discomfort. This value continues to be used as the initial criterion for using magnetic fields to apply heat to patients [34], [47]. Assuming a typical frequency of 100 kHz and a specific heat capacity of 0.1 W/ml [8], [9], [15]–[18], [22], [24], [25], [28], [47], a magnetic field of 4850 A/m or 6.1 mT is obtained according to the Atkinson and Brezovich limit. If the solution is Resovist®, then a value of 8.55 mgFe/ml or 0.855% is obtained for *D_w_*. This value should be considered as an indication rather than an absolute limit. In fact, all the values used for this calculation should not be considered as a substitute for the proper measurement of clinical tolerability under therapeutic conditions. The temperature increase over a period of time is an essential parameter in MFH. However, the increase does not solely depend on *Q*; several other clinical factors, such as the heterogeneity of the tumor and surrounding tissue, the size and pH of the tumor, blood flow in the tumor and surrounding areas, as well as heat radiation influence the temperature increase.

**Figure 1:**
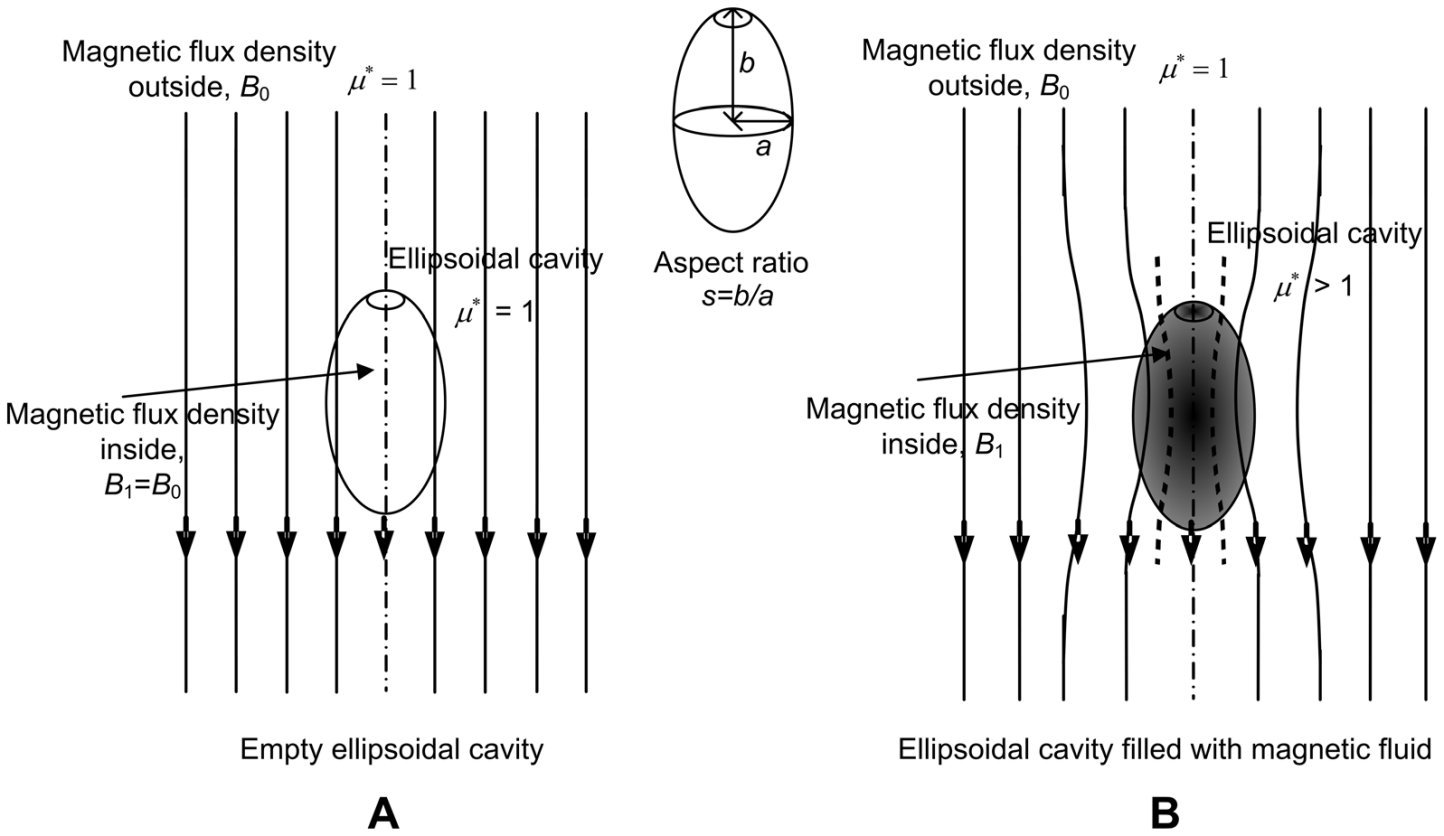
Analytical model for estimating magnetic fluid weight density inside a tumor. Magnetic flux density inside and outside a magnetic fluid-filled ellipsoidal cavity. The flux lines pass through the empty cavity (A) but converge in the cavity with magnetic fluid (B), thus leading to a difference in the magnetic flux density inside and outside the cavity.

Consider the situation shown in Figure 1. An ellipsoidal cavity filled with magnetic fluid is placed under a uniform magnetic flux density. Given that the outside environment is air with μ* = 1, and that the magnetic fluid has μ* slightly greater than 1, magnetic flux lines will converge and concentrate at the magnetic fluid filled ellipsoidal cavity. If a magnetic flux density, *B*_0_, is applied then the magnetic flux density in the cavity, *B*_1_ will change according to *D_w_*. *B*_1_, can be expressed according to the following equations [48]. 
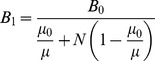
(2)

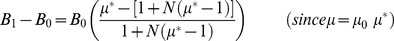
(3)

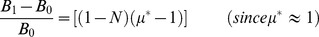
(4)where 
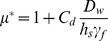
 is based on permeance calculations [42]; *h_s_* is the space factor for spherical magnetite (0.523), *γ_f_* is the specific gravity of magnetic fluid (4.58) and *C_d_* is a coefficient (theoretically 4).

The basis for estimating *D_w_* is the difference in magnetic flux density inside, (*B_1_*), and outside, (*B_0_*), a tumor, when the tumor is under the influence of a uniform magnetic flux density. Substituting 
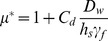
 into (4) and for *D_w_*<<1, the change in magnetic flux density (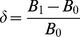) is given by 
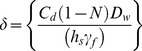
(5)


The most important points to be observed from equation (5) are that the change in the magnetic flux density is proportional to the weight density, but independent of the shape and/or size of magnetic nanoparticles in the magnetic fluid. However, the shape of the cavity enclosing the magnetic fluid influences *δ*; this is expressed in equation (5) as the demagnetizing factor (*N*). *N* depends on the aspect ratio of the cavity, *s*; for an ellipsoidal cavity *s*  =  major axis *b*/minor axis *a*, as shown in Figure 1 [49]. During tumor heating in MFH therapy, the magnetic fluid spreads after being injected which means that μ* and *D_w_* are bound to vary inside the tumor as opposed to being uniform, thus forming the basis for this research.

### 2. A Numerical Model to Analyze the Distribution of Magnetic Fluid inside Tumors

In the previous section a relationship was obtained between *δ* and *D_w_*, assuming that the magnetic fluid distribution was uniform inside the ellipsoidal cavity. However, in a realistic clinical situation, the magnetic fluid most likely spreads inhomogeneously inside the tumor. Therefore, numerical analysis was performed to analyze the distribution of *D_w_* inside tumors, taking into account the analytical analysis based on ellipsoidal cavities. The tumor model that was used in the numerical analysis was based not only on the analytical analysis, but also on the feasibility of building such a model experimentally and utilizing the GMR for analyzing *D_w_* inside tumor models. The shape of the tumor modeled numerically was cylindrical. Even though tumors are assumed to be spherical in most studies, we chose cylindrical cavities instead because spherical cavities are difficult to make using agar or other materials used for making experimental phantoms; the *N* for cylindrical cavities (0.3116) is very close to the *N* for spherical cavities (0.33) [50].

A two-dimensional finite element model (FEM) of a double-cavity tumor was simulated in COMSOL® to obtain numerical results for *δ* in a cylindrical container filled with different concentrations of magnetic fluid. The model was meshed with ‘Lagrange-Quadratic’ elements, meaning that the solution was approximated with second degree polynomials. A Quasi-statics analysis was carried out; an approximation that can be considered valid given that the frequency was 100 Hz, and that the model was considerably smaller than the wavelength.

**Figure 2:**
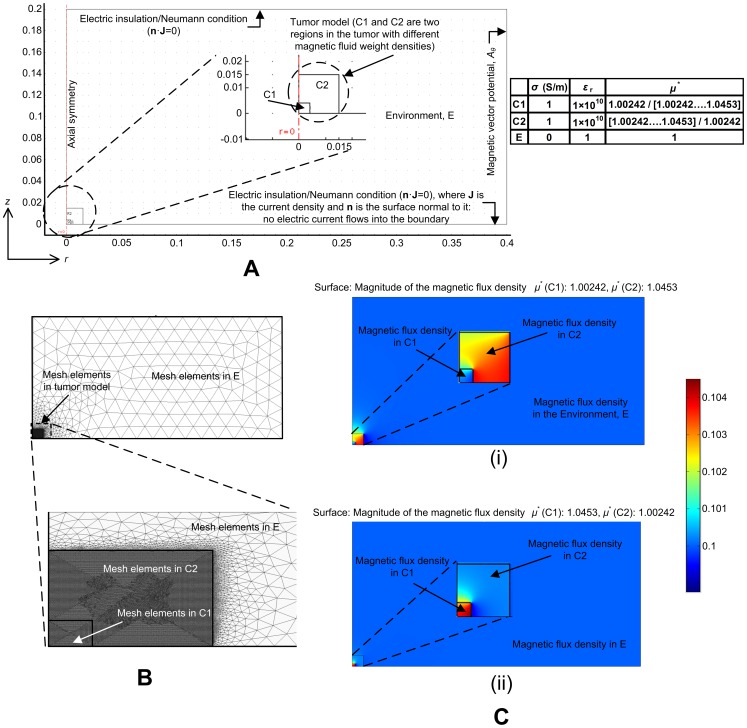
Numerical modeling. (A) 2-D, axial-symmetric model, (B) meshed model, and (C) solved model for the cases (i) *D_wi_* < *D_wo_* and ii) *D_wi_* > *D_wo_*.

The numerical model is shown in Figure 2 (A). Exploiting the symmetry of cylinders about their central axis, and about a plane through their center, only a quarter section of the cylinder was modeled. There are two cylindrical cavities, cavity 1 (C1) and cavity 2 (C2), enclosed in an environment (E); C1 and C2 represent two regions inside a tumor, each having a different *D_w_*, corresponding to the non-uniformity of the tumor. The side lengths of C1 and C2 are 4 and 15 mm, respectively; when revolved in 3-D to a full cylinder these side lengths correspond to a diameter and height of 8 mm for C1 and 30 mm for C2. The *r*-axis is parallel to the diameter of the cylinder and the *z*-axis is perpendicular to the diameter of the cylinder. The subdomain and boundary settings are shown in Figure 2 (A). An electrical conductivity (*σ*) value of 1 S/m and a relative permittivity (*ε_r_*) value of 1×10^10^ was assumed for C1 and C2 (agar and magnetic fluid media) based on [51]–[60]. E was assumed to be air, so *σ* was set to 0 S/m and *ε_r_* to 1. Two simulations were performed, one for the case where *D_w_* inside C1 (*D_wi_*) was less than the *D_w_* inside C2 (*D_wo_*) and another for the case where *D_wi_* > *D_wo_*. For the case *D_wi_* < *D_wo_*, μ* in C1 was set to 1.00242 and μ* in C2 was set to [1.00242, 1.01359, 1.01889, 1.02742, 1.03540, 1.04143, 1.0453], and for the case *D_wi_* > *D_wo_*, μ* in C1 was set to [1.00242, 1.01359, 1.01889, 1.02742, 1.03540, 1.04143, 1.0453] and μ* in C2 was set to 1.00242. The boundary of the surrounding environment was considered electrically insulating (Neumann condition) because in the experimental situation the magnetic flux lines will continue to infinity. The common boundary between the two cavities and the environment was set to ensure axial symmetry, and the boundary opposite to this was given a magnetic potential. The uniform magnetic flux density and the magnetic vector potential *A* are related as follows: 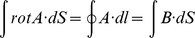
(6)


For an axially symmetric model of radius *r*, *A* is the *θ*-axis component and *B* is the *z*-axis component. Therefore, 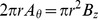
(7)


Then, 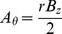
(8)


The applied magnetic flux density *B* (*B*_0_) was set to 0.1 mT; the reason for choosing this value was because the mid-point of the operating region of the sensor (the maximum dc sensitivity-linear region) was approximately 0.1 mT. Substituting *B* = 0.1 mT and *r* = 0.4 m (radius of the model) into equation (8), *A_θ_* was calculated to be 0.02 Wb/m.

Figure 2 (B) shows the discretized model consisting of 64517 elements. C1 and C2 had more refined meshes than E because the analysis of magnetic flux distribution was studied in detail in these regions; C1, C2 and E had 4040, 55774 and 4703 mesh elements respectively. Figure 2 (C)-(i) shows the solved model for *D_wi_* < *D_wo_* and Figure 2 (C)-(ii) for *D_wi_* > *D_wo_*. Figure 2 (C)-(i) demonstrates that the magnitude of *B* in C1 is lower than C2, and Figure 2 (C)-(ii) demonstrates that *B* is higher in C1 than in C2, thus corresponding to the *D_w_* values in C1 and C2. These models were used to obtain numerical results for *δ* as a function of *r* and *D_w_*; *B*_1_ (*r*, 0) corresponded to the sensing signal, while *B*_0_ (*r*, 20 mm) corresponded to the applied magnetic flux density outside the tumor model. The results obtained were used to introduce a basic methodology to analyze the magnetic fluid distribution inside a tumor that has potential to be expanded to tumors with many areas of non-uniformity (a multi-cavity model), which is quite possibly encountered regularly in clinical MFH treatment.

### 3. GMR Probe

GMR sensors have several advantages over other magnetic sensors when utilized for biomagnetic measurements; GMR sensors require only a small *B* to change their resistance (highly sensitive to minute changes in magnetic flux densities), and have advantages with respect to size, cost, power and thermal stability compared to commonly used magnetic sensors such as SQUID, Hall, search coil, or fluxgate sensors [61]–[67]. Furthermore, GMR sensors are easily energized by applying a constant current and the output voltage is a measure of *B*, so they are ideal for low cost applications.

**Figure 3:**
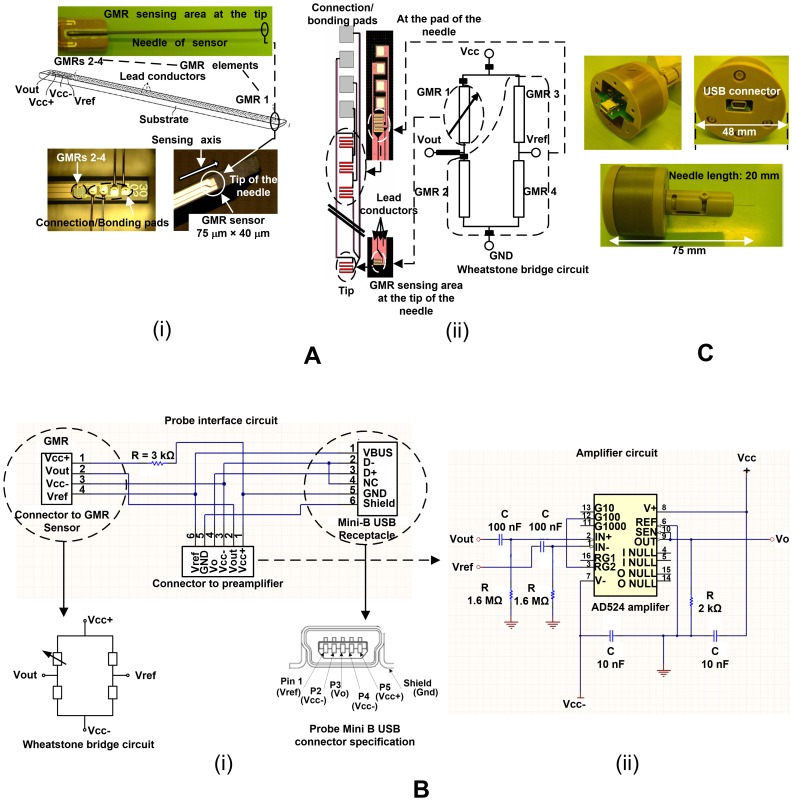
The giant magnetoresistance probe. (A) (i) Needle sensor, and (ii) Wheatstone bridge design of the sensors. (B) (i) Probe interface circuit, and (ii) amplifier circuit. (C) Fabricated probe with the USB connector.

The GMR probe that was designed and fabricated has a needle-shaped detecting part as shown in Figure 3 (A)-(i). The length and diameter of the needle are 20 mm and 310 µm, respectively. By having a sensor on the needle, the probe can be made compact, and in the event that a detection element is attached to the needle, the influence due to the shape of the substrate can be reduced to a certain extent. Aluminum titanium carbide, a sintered material of aluminum oxide and titanium carbide, was used as the base material to make the needle mechanically strong, since such a fine needle could be expected to break easily due to a lack of rigidity. The needle-shaped detecting part consists of a substrate (to which a machining process was applied in order to cut it to a needle shape), four spin valve GMR elements formed of thin films on the surface of the substrate, four connection/bonding pads, lead conductors for electrically connecting the spin valve GMR elements to the connection/bonding pads, and a protection film for covering the spin valve GMR elements and lead conductors, except parts of the connection/bonding pads. The connection/bonding pads were formed by a bump layer of Cu, and a bonding pad layer of Au that was laid on the bump layer. Diamond like carbon was used as the surface modifying layer since it is biocompatible.

The spin valve GMR sensors, connection/bonding pads and lead conductors were formed on a wafer by a wafer process utilizing thin film photolithography techniques. The needle has a sensing GMR (GMR 1) element at the tip and reference GMR elements (GMRs 2–4) at the root near the connection/bonding pads as shown in Figure 3 (A)-(i). The GMR sensing elements were connected in a Wheatstone bridge circuit to reduce bias and noise signals as shown in Figure 3 (A)-(ii). Due to limitations in the fabrication process it is very difficult to obtain exactly the same resistances for the sensing elements, giving rise to an offset in the bridge circuit. To compensate for this imbalance the lengths and widths of the lead conductors connecting the sensing elements were adjusted accordingly, as shown in Figure 3 (A)-(ii). The sensing GMR element measures the magnetic flux density inside the tumor, while the reference elements are exposed to the applied magnetic flux density; this enables the probe to estimate *B*_0_ and *B*_1_ simultaneously. The bridge output was connected to a preamplifier (AD 524 precision instrumentation amplifier) inside the probe, which in turn was connected to a Mini B USB connector, as shown in Figure 3 (B)-(i). There are two high pass filters on the differential inputs to the AD524 (IN+ and IN-) to eliminate signals at very low frequencies (cutoff frequency of 1 Hz), and the amplification was set to 100V/V (40 dB), as shown in Figure 3 (B)-(ii). Two bypass capacitors (10 nF) were used to dampen AC components and noise. The fabricated probe with the USB connector is shown in Figure 3 (C). The casing that houses the needle and the flexible printed circuit board was made from UNILATE™ of UNITKA, which is a polyethylene telephthalate composite resin. However, as a general rule, the case must be made of any material that is non-magnetic due to the high sensitivity requirements for low magnetic flux measurements. When the tip of the needle is inserted into a magnetic medium, the resistance of the sensing GMR element changes, triggering an output voltage at the Wheatstone bridge. This enables the calculation of *δ*, which is correlated to *D_w_*.

### 4. Experimental Setup

Agar powder with a jelly strength of 400–600 g/cm^2^ by Wako Company was used to make experimental tumor models. To make pure solidified agar, the concentration requirement was 0.5 g agar powder per 100 ml distilled water. The agar powder was mixed with distilled water at boiling or near boiling temperatures. Once the powder was melted and mixed well with distilled water, the agar/water complex was placed in the refrigerator to solidify. Cylindrical agar models, with diameters and heights of 8 and 30 mm (*N* = 1), respectively, were made to simulate tumors with two cavities. The cylindrical agar models were injected with various weight densities of a water-based magnetic fluid (Taiho industries Co. Ltd, original weight density 40% (40 mg/ml)) to simulate magnetic fluid filled tumors. The magnetic fluid weight densities used in the experiments (0.814%–2.713%) were chosen for two reasons: i) Even though magnetic fluids like Resovist® used in MFH are clinically approved, surgeons still prefer to perform frequent, repeated treatments with low densities (generally <2.8%), and ii) the fluid density may very well decrease once injected into the tumor by spreading to neighboring tissues, and a fraction of this density may still remain after treatment. Therefore, it is important to be able to detect concentrations much smaller than 2.8%. Moreover, the lower the weight density that can be detected and estimated, the wider the area of distribution that can be mapped by the GMR probe. It follows that if the GMR probe is sensitive enough to detect and estimate such low weight densities, it would naturally be able to detect and estimate higher weight densities, if needed.

**Figure 4:**
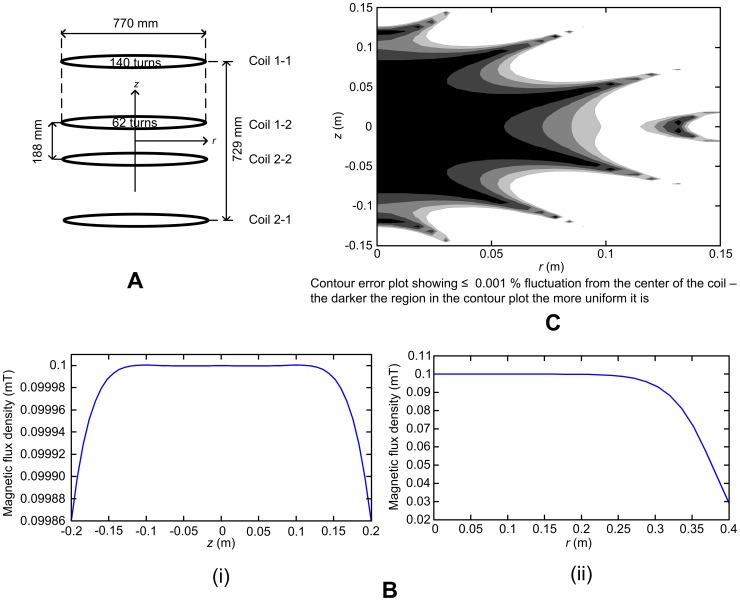
The Lee-Whiting coil. (A) Coil design, (B) (i) magnetic flux distribution in the *z*-direction, (ii) magnetic flux density distribution in the *r*-direction, and (C) fluctuation of the magnetic flux density from the center of the coil.

A uniform magnetic flux density is an important requirement for the estimation of *D_w_* when using the probe. A Lee-Whiting type coil [68] was designed and fabricated, producing a 0.001% fluctuation from the center of the coil in approximately 35% of the outer coil spacing along the *z*-direction and 25% of the diameter of the coils in the *r*-direction. The design of the Lee-Whiting coil is shown in Figure 4 (A) and the analytical results are shown in Figure 4 (B–C). The distance between the active GMR sensor at the tip and the reference GMR sensors near the pads is approximately 20 mm. Ideally, the tip of the active GMR sensor is at the center of the Helmholtz coil, and the GMR reference sensors are 20 mm above the Helmholtz coil in the *z*-direction. The fluctuation of *B*, calculated at 20 mm from the center of the designed coil, is 2.6×10^−5^%. The magnetic flux density change due to *D_w_* is required to be higher than this fluctuation in order to obtain reliable data.

**Figure 5:**
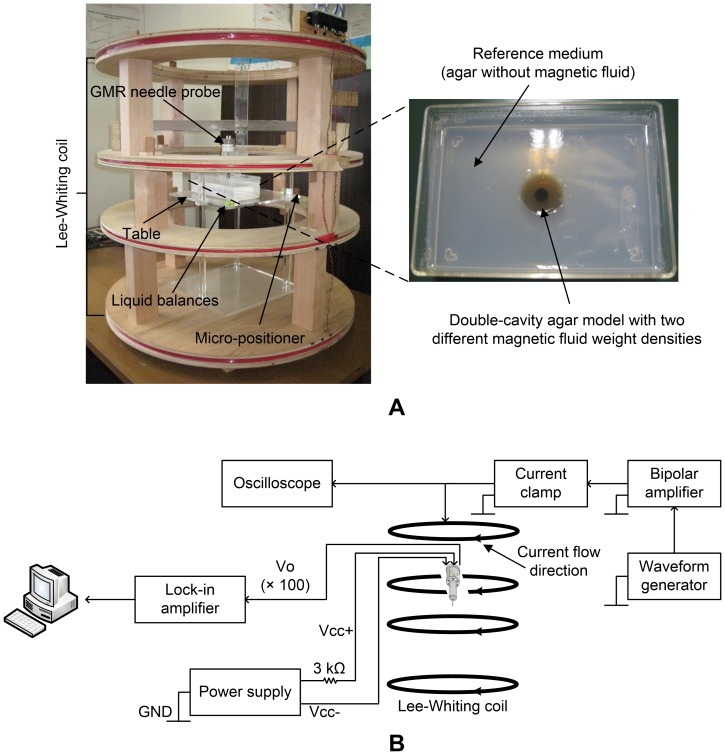
(A) Experimental setup, and (B) schematic of the data analysis setup.

The experimental setup is shown in Figure 5 (A). The experiments were performed in an isolated room where only the instruments used in the experiments were present. Proper alignment of the experimental setup was ensured by using liquid balances. Using a micro-positioner, the needle was precisely placed at analysis points of the cavity for various values of *r*. The output (Gain = 100) of the instrumentation amplifier was then fed into a digital lock-in amplifier (NF Electronics LI5640) as shown in Figure 5 (B). The lock-in amplifier was used to measure the signal at 100 Hz with a bandwidth of 1 Hz. A value was obtained as an average of five readings taken at 5 second intervals once the signal settled after 60 s.

## Results and Discussion

### 1. Numerical Results of the Magnetic Flux Density Distribution inside Cylindrical Cavities

**Figure 6:**
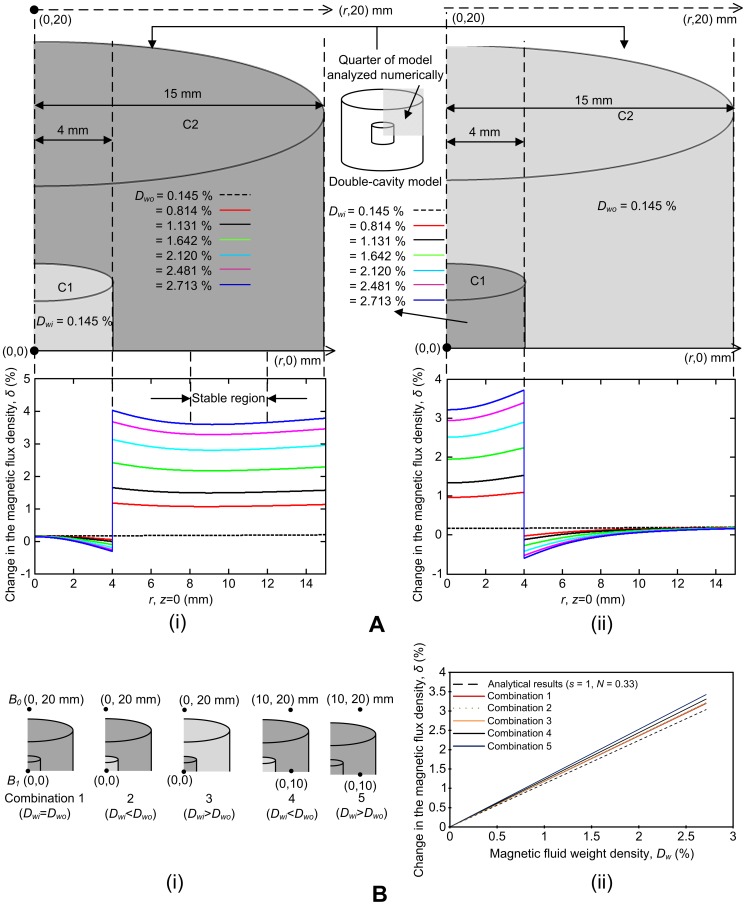
Numerical results. (A) The magnetic flux density distribution when (i) *D_w_*_i_ < *D_w_*_o_, and (ii) *D_wi_* > *D_wo_*. (B) (i) Different combinations of magnetic fluid weight densities to be estimated and surrounding media with varying weight densities. (ii) Comparison of magnetic fluid weight densities for surrounding mediums with different weight densities.

The numerical analysis performed showed a clear change in *δ* when moving along the (*r*, 0) axis, from one cavity with a density *D_w1_* to another cavity with a density *D_w2_*. This change was most significant when *δ* was obtained along the radial (*r*) axis while the axial (*z*) axis was 0. In the numerical model, both the inner and outer cavities were centered at (0,0) mm. As shown in Figure 6 (A)-(i), there is a significant increase in the magnetic flux density values when moving from the inner cavity (C1) to the outer cavity (C2). This increase is proportional to the *D_w_* values. For example, the highest change is observed for *D_wo_* = 2.713% and the lowest for *D_wo_* = 0.814% (there is no significant change when *D_wo_* is 0.145% since then *D_wo_*  =  *D_wi_*). These first two observations concur with equation (5), which shows that *δ* is proportional to *D_w_*. In addition, *δ* is relatively stable from *r* = 8–12 mm, between the outer boundaries of the two cavities (*r* = 4 and 15 mm). Therefore, in the outer cavity, *D_w_* can be estimated anywhere along *r* = 8–12 mm. For the case when *D_wi_* is higher than *D_wo_*, there is a significant decrease in the *B* values when moving from C1 to C2, and this decrease, as for the case when *D_wi_* is lower than *D_wo_*, is proportional to the *D_w_* values (Figure 6 (A)-(ii)). However, since the boundaries of C1 are extremely close to each other (4 mm compared with 11 mm in Figure 6 (A)-(i)), the most suitable point for estimation is at the center of the tumor model, at (0,0). Therefore, the GMR probe can be utilized to obtain signals corresponding to changes in magnetic flux densities by inserting the needle into these points (at the center (0,0) of the inner cavity and at a point in the stable region of the outer cavity).

Next, several tumor models with different combinations of weight densities and analysis points were considered in order to investigate how values, estimated at a point in the stable region and at the center of the tumor model, compare with analytical values based on ellipsoidal cavities. In Figure 6 (B)-(i), combination 1 shows a cylindrical cavity where *D_wi_* equals *D_wo_* and the weight density is estimated at the center of the cavity. In combinations 2 (*D_wi_* < *D_wo_*) and 3 (*D_wi_* > *D_wo_*), the weight density is also estimated at the center of the cavity. However, in combinations 4 (*D_wi_* < *D_wo_*) and 5 (*D_wi_* > *D_wo_*), the weight density is estimated at 10 mm from the center of the tumor model at (0,10) mm; this point is in the stable region depicted in Figure 6 (A)-(i). Figure 6 (B)-(ii) shows the numerical results obtained for these five combinations compared with a spherical cavity (*N* = 0.33). For all combinations, *δ* is proportional to *D_w_*. Also, since *N* is lower for cylindrical cavities (0.3116) than for spherical cavities (0.33) the trendlines for all five combinations are higher than the analytical line (see equation (5)). Numerical results for combinations 2 and 3 are very close to combination 1. Combination 1 is the ideal case, where there is no distribution of magnetic fluid and *D_w_* can thus be estimated at the center of the tumor model. Although *D_w_* is estimated at the center of the cavity for both combinations 2 and 3, the tumor model does not have a uniform distribution of *D_w_*; *D_wi_* and *D_wo_* are different inside the two cavities. However, when *D_w_* is estimated at the center, the result is very close to the numerical results This observation leads us to conclude that if there were a given volume of tumor in the middle with a certain weight density, surrounded by lower or higher weight densities, the GMR probe would quite possibly still be able to measure *B* at the center of the tumor and estimate *D_w_* by the difference between the flux densities inside and outside the tumor. Combinations 4 and 5 showed the highest variance in the change in magnetic flux densities compared with the numerical results, since *B* was estimated at a point in the stable region (0,10) mm and not at the center of the tumor model (0,0). *B* is most uniform at the center of the tumor model and dominant in the *z*-direction. The larger change in magnetic flux densities observed for combinations 4 and 5 can be attributed to the decrease in the uniformity of the magnetic flux density values along the *r*-axis; flux density components in directions other than the *z*-direction decrease the magnitude and uniformity in the *z*-direction.

Two important points can be gathered from the numerical results: i) There is a significant difference in the change in the magnetic flux density proportional to *D_w_* in tumor models with two cavities, where each cavity has a different *D_w_*, and ii) this change in magnetic flux density compares well with analytical results, leading to a firm basis for experimental analysis. Hence, this methodology can be used to not only detect but also to estimate *D_w_* in a double-cavity tumor model.

### 2. Experimental Analysis

**Figure 7:**
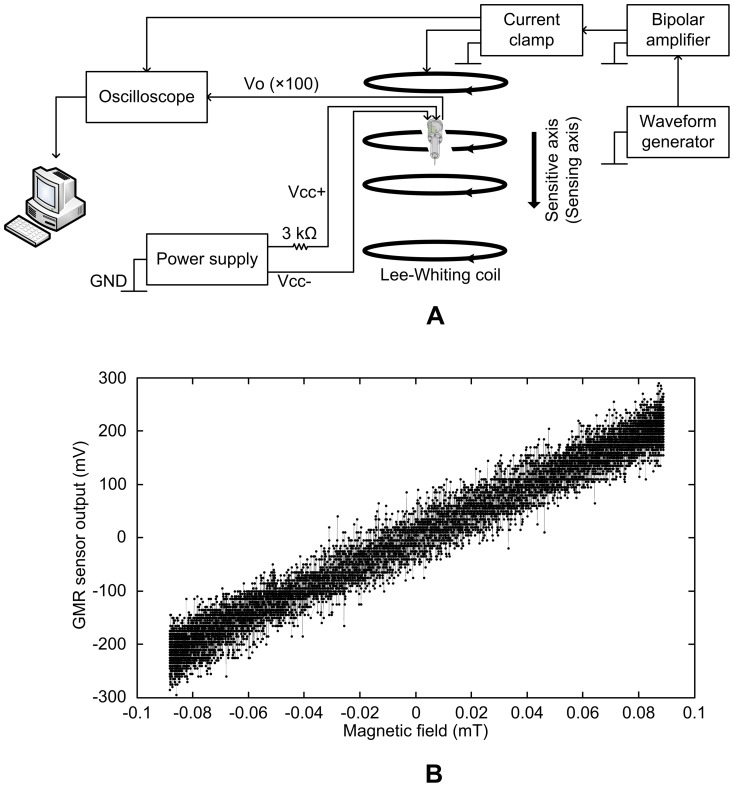
GMR probe characterization. (A) Experimental setup for characterization, (B) small-signal AC sensitivity at 100 Hz.

A small signal AC sensitivity characterization of the GMR probe was carried out for the sensing GMR element (GMR 1) along the sensitive axis of GMR 1 as shown in Figure 7 (A). The Lee-Whiting coil was used to apply a uniform *B* of 0.09 mT at a frequency of 100 Hz. The coil was driven by a sinusoidal current provided by a function generator (Sony Tektronix AFG310) connected to a high-speed power amplifier (NF Electronics 4055). The probe output and the output of a current clamp (Hioki 3274), measuring the current in the coil, were connected to an oscilloscope (Yokogawa DL4100). The waveforms in the oscilloscope were transferred to a computer by a general purpose interface bus (GPIB) to evaluate the AC sensitivity. The 100 times amplified small signal AC sensitivity of the GMR sensor was approximately 2800 mV/mT as shown in Figure 7 (B).

**Figure 8:**
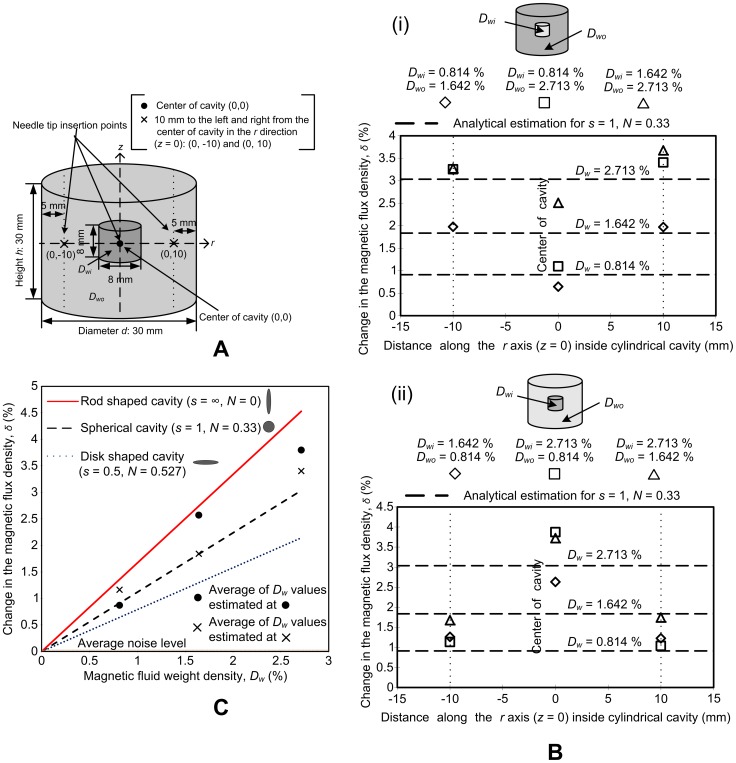
Experimental analysis. (A) Schematic of the double-cavity cylindrical agar tumor model. (B) Magnetic fluid distribution analysis in double-cavity agar models. (i) *D_wi_* < *D_wo_* and (ii) *D_wi_* > *D_wo_*. (C) Correlation of experimental data with analytical results.

The main objective of the experimental analysis was to observe if the GMR probe could be used to analyze the distribution of two magnetic weight densities in different areas of a single agar tumor model. Figure 8 (A) shows the schematic of the double-cavity agar tumor model. The dimensions of the model are the same as the model used for numerical analysis. Furthermore, the needle tip insertion points are based on the numerical analysis and placed at the center of the model (0,0) and 10 mm to the right (0,10) and left (0,−10), of the center. The position along the *z*-axis is 0. The distance between the GMR sensor at the tip and the references sensors is 20 mm. Since the height of the tumor model from the top to the center is 15 mm, the reference sensors are 5 mm above the top of the tumor model, when the needle is at the center; therefore, the reference sensors are exposed to the applied *B*. In this way the GMR probe is able to measure both the magnetic flux density inside the cavity (*B*_1_) and outside the cavity (*B*_0_) simultaneously.

Initial experiments were performed with agar tumor models without magnetic fluid to observe the response of the GMR probe. Ideally, the differential signal would be 0, since the relative permeability of agar and air is 1; therefore, both *B*_1_ and *B*_0_ should be the same. However, an average change in magnetic flux density of 0.03% was observed. This change in magnetic flux density corresponds to a *D_w_* of 0.0268%, the limit of resolution of the current setup.

Figure 8 (B)-(i) shows the experimental results for a double-cavity agar model where *D_wi_* is lower than *D_wo_*. Three *D_w_* values were used in three different mixtures. In mixture 1 (denoted by diamond data points), *D_wi_* is 0.814% and *D_wo_* is 1.642%; in mixture 2 (denoted by square data points), *D_wi_* is 0.814% and *D_wo_* is 2.713%; and in mixture 3 (denoted by triangular data points), *D_wi_* is 1.642% and *D_wo_* is 2.713%. Note that the differences in the change in magnetic flux densities for all these mixtures are more than the average noise signal of 0.03%. The GMR probe was inserted at (0,−10) mm, then (0,0) mm, and finally at (0,10) mm. The experimental results demonstrate a significant change in *δ* for all three mixtures when the probe was moved through the agar model. *δ* decreased when the needle was moved from (0,−10) mm to (0,0) mm, because *D_w_* at (0,−10) mm is higher than *D_w_* at (0,0) mm, and then it increased when the needle was subsequently moved to (0,10) mm because of the higher *D_w_* there, giving rise to a ‘trough’ shape. The change is also proportional to the change in *D_w_*. For example, the change in *δ* for mixture 2 is highest because the difference in *D_w_* is the highest (2.713%−0.814% = 1.8990%). The decrease in *δ* is not so clear-cut for mixtures 1 and 3, because the difference in their *D_w_* values is very small (0.8280% for mixture 1 and 1.0710% for mixture 3). Figure 8 (B)-(ii) shows the results for a double-cavity tumor model with the same three mixtures as in Figure 8 (B)-(i), but for the case when *D_wi_* is greater than *D_wo_*. The experimental results show a significant change in *δ* and, as expected, the behavior is opposite to that shown in Figure 8 (B)-(i), since *D_w_* is highest at (0,0) mm, giving rise to a ‘peak’. Again, the changes in *δ* are highest for mixture 2, because it has the largest difference in the magnetic fluid weight density (*D_wi_*-*D_wo_*) and the change is not so obvious for mixtures 1 and 3. All the data points in Figure 8 (B) were also compared to *δ* values obtained by analytical analysis (dashed lines in Figure 8 (B)). It can be seen that the measured points do not deviate greatly from the analytical lines; a deviation corresponding to the difference in the demagnetization values between a spherical and cylindrical cavity is expected.

In general, inside a uniformly magnetized ferromagnetic body (μ*>1), *B* is not uniform. The only geometric shape in which it is uniform in practice is an ellipsoid. In all non-ellipsoidal shapes, the demagnetizing factor *N*, is used to approximate the internal magnetic flux densities. Most studies have assumed that tumors are spherical because they are easiest to examine theoretically. For a spherical tumor, *s* = 1 and *N* = 1/3; if a tumor is long and thin (rod-shaped), *s*  =  ∞ and *N* = 0; and if a tumor is short and flat (disk-shaped), *s* = 0.5 and *N* = 0.527 [49]. In Figure 8 (C), the values for a rod-shape are used as the upper limit of error and those for a disk shape as the lower limit of error. These limits are for cavities that have a uniform magnetic fluid distribution and hence a uniform permeability inside (*D_wi_*  =  *D_wo_*), and *δ* is calculated at the center of the cavity. The magnetic fluid weight densities detected inside the agar cavities in Figure 8 (B) are shown in the graph. These points are the average of data values at each point in the cavity in Figure 8 (B). The average values estimated at the center are denoted by •, and the values estimated at (0,−10) mm and (0,10) mm are denoted by **×**. The average signal, when no magnetic fluid is present in the agar cavities, is also added to the graph and can be considered as the average noise signal. As can be seen from Figure 8 (C), all the experimental results fall within the upper and lower limits of *N*. This shows that unless the tumor has a highly irregular shape, the proposed methodology provides a sound basis for estimating magnetic fluid weight densities inside tumor formations.

### 3. Discussion

The average noise signal observed when there was no magnetic fluid present in agar cavities has several sources. Besides electromagnetic and instrumentation noise, the Lee-Whiting coil may be the dominant contributor. An analysis was performed to show how errors in coiling and construction could affect the uniformity of the applied magnetic flux density. With reference to Figure S1 (A), coils 1–1 and 1–2 can be reasonably expected to shift up to +/− 2 mm in the *r*- and *z*-directions during assembly. The errors at 20 mm in the *z*-direction (the distance between the active and reference GMR sensors) due to these shifts are shown in Figure S1 (B). It can be seen that the fluctuations increase 100 fold for +/− 1 mm shifts and 1000 fold for +/− 2 mm shifts. Moreover, if the current distribution is considered to be a square (due to actual coiling) instead of a point (as assumed in the analytical analysis), the error increases to 4×10^−5^%.

The reason for fluctuations in Figure 8 (B) and (C), which are larger than the fluctuations due to the difference in *N* values for a sphere and cylinder, is most probably due to errors in positioning of the needle tip, since *B* is highest at the center and decreases in both positive and negative directions along the needle insertion axis (the *z*-axis). Another plausible reason is that remainders of magnetic fluid accumulate at the needle, resulting in a cumulative effect with each insertion, even though the sensor was dipped in alcohol after each insertion to clean the agar and magnetic fluid mixture.

If the GMR needle probe is to be used *in vivo* it is also important to consider bodily fluid and tissues that are conductive, and the implications caused by exposing them to high frequency fields. Therefore, it is important to perform analysis at very low AC frequencies since high-frequency magnetic flux can induce eddy currents on conductive media in the body and the resulting electromagnetic fields have the possibility of modifying the GMR needle probe measurements (please see Figure S2). No discernible difference was observed in the *B* distribution at frequencies of 100 and 1000 Hz (Figures S2 (A)-(i) and (ii) and Figures S2 (B)-(i) and (ii)), and the distributions in both cases were comparable to the numerical results obtained in Figures 6 (A)-(i) and (ii) for *σ* = 1 S/m. However, the effect of eddy currents was seen at 10000 Hz (Figure S2 (A)-(iii) and Figure S2 (B)-(iii)).

The cavities used to simulate cancerous tumors in this research were limited to cylindrical shapes. Currently, it is assumed that tumors are spherical in shape; however, in reality they could be any shape. The orientation of the cavity should also be considered. Cylindrical cavities used for the experiments are symmetrical in the *r*- and *z*-directions, but tumor growth inside the body can be in any direction. Hence, it is necessary to perform more experiments with different shapes and orientations of cavities.

## Conclusion

This paper investigated the feasibility of analyzing the distribution of magnetic fluid, as used in MFH therapy, utilizing a GMR probe. Once the distribution of the fluid is known, the magnitude and frequency of the applied magnetic flux density can be tuned to optimize the effects of MFH therapy. The key feature of this research is the GMR probe that was designed and fabricated to be inserted into the human body in a minimally-invasive way. Small-signal AC characterization showed that the GMR probe has a sensitivity of 2800 mV/mT. An analytical model was presented to estimate magnetic fluid weight density (*D_w_*) inside tumors using the GMR probe. The difference in the magnetic flux density inside (*B_1_*) and outside (*B_0_*) a magnetic fluid-filled cavity, under the influence of a uniform magnetic flux density, was quantified by numerical analysis. *B_1_* and *B_0_* were then expressed in terms of the relative permeability and *D_w_* of the magnetic fluid, leading to a method of estimating *D_w_*. The needle of the GMR probe has an active GMR sensing element at the tip and three reference GMR sensing elements 20 mm further up the needle. This unique design allows it to measure both *B*_1_ and *B*_0_ simultaneously. Double-cavity agar tumor models were made with different values of *D_w_*. Three magnetic fluid weight densities (0.814%, 1.642% and 2.713%) were tested in three combinations. The *D_w_* values tested were less than 2.8%, which corresponds to typical values used in clinical applications. A Lee-Whiting coil was designed and fabricated to provide a uniform magnetic flux density of 0.1 mT at a frequency of 100 Hz. The experimental results showed that the GMR probe was able to detect different weight densities inside the agar tumor model, and that *D_w_* was proportional to the change in magnetic flux density (*B*_1_ − *B*_0_). The experimental results corresponded well with analytical and numerical results; the higher the difference between the magnetic fluid weight densities, the higher the difference between the changes in magnetic flux densities obtained by the GMR probe. The results were also within the error limits due to the shape of the cavity, considering rod-shaped and disk-shaped cavities. This indicated that the magnetic fluid weight density can be estimated by the GMR probe inside tumors with a wide range of different shapes. Based on the average noise signal of 0.03%, which is the change in the magnetic flux density for agar cavities without any magnetic fluid, the limit of *D_w_* that could be estimated with the current setup is 0.0268%. The experimental results obtained in this paper suggest the possibility of extending and using this methodology for obtaining magnetic fluid weight density maps in tumors with an inhomogeneous distribution of magnetic fluid.

## Supporting Information

Figure S1 **(A) Possible errors due to construction and coiling.** (B) Fluctuations in the magnetic flux density at 20 mm in the *z*-direction from the center of the coil system, when the radius or the distance between coils 1–1 and 1–2 is shifted 2 mm.
(TIF)

Figure S2 **Influence of eddy currents on the distribution of magnetic flux density.** (A) Magnetic flux density distribution when *D_wi_* < *D_wo_*, at frequencies of (i) 100 Hz (ii) 1000 Hz and (iii) 10,000 Hz. (B) Magnetic flux density distribution when *D_wi_* > *D_wo_*, at frequencies of (i) 100 Hz (ii) 1000 Hz and (iii) 10,000 Hz.
(TIF)
